# Clinical Diagnosis and Reporting of COVID-19 in the Absence of Effective Access to Laboratory Testing in Africa

**DOI:** 10.3389/fpubh.2021.645200

**Published:** 2021-05-28

**Authors:** John Walley, Akaninyene Otu, Emmanuel Effa, Laura French, Obiageli Onwusaka

**Affiliations:** ^1^Leeds Institute of Health Sciences, Nuffield Centre for International Health, University of Leeds, Leeds, United Kingdom; ^2^Department of Internal Medicine, Faculty of Medicine, College of Medical Sciences, University of Calabar, Calabar, Nigeria; ^3^Foundation for Healthcare Innovation and Development (FHIND), Calabar, Nigeria; ^4^Department of Public Health, Faculty of Allied Medical Sciences, College of Medical Sciences, University of Calabar, Calabar, Nigeria

**Keywords:** COVID-19, testing, Africa, clinical diagnosis, clinical algorithm

## Introduction

In reality, in most of sub-Saharan Africa, either COVID-19 tests (1) are unavailable or turnaround time is long or results take too long to return from distant laboratories (2). Even for tests now done at local laboratories, the turnaround time is longer because of the volume, frequent stockouts of reagent and sample collection kits, and power cuts. For example, in Cross River State in South-Eastern Nigeria (population 3.7 million) as of December 13, 2020, only 1993 COVID-19 RT-PCR tests have reportedly been performed since the pandemic onset (at centralized sites of the National Centre for Disease Control) (3). This situation suits the politico-economic drivers to ease lockdown restrictions, but creates a false sense of security. Some symptoms of COVID-19 overlap with those of common infections such as malaria, dengue, and pneumonia, making diagnosis challenging without a test.

## How Accurate are COVID-19 RT-PCR Tests?

The COVID-19 RT-PCR test has a sensitivity of 71–98% (4), equating to false-negative rates of 2–29% and high specificity, though accuracy of testing is dependent on the quality of sampling and when in the course of illness the sample is collected. It is difficult for clinicians (and more so for the public) to understand how COVID-19 RT-PCR tests could give false-negative results—especially in week 1 of the illness (5) with grave consequences if people then fail to follow preventive measures.

The question then arises, “How can diagnosis of COVID-19 be improved in resource-constrained settings with limited diagnostic testing capacity?” The answer is that there is an urgent need instead for a clinical diagnostic algorithm (based on symptoms, available laboratory tests and chest X-rays). Such an algorithm will guide decisions to treat and give preventive advice including home isolation and quarantine of close family/contacts. Ideally, this tool would also have sensitivity and specificity close to that of the RT-PCR test in week 1 of COVID-19 disease. The algorithm should include asking for symptoms (questions about loss of taste/smell in addition to other more diverse symptoms, contact and co-morbidities), assessing severity of the disease (using CRB65 or NEWS score), identifying suspected cases, and prescribing appropriate care (Appendix 1).

## Predictive Value of Clinical Symptoms for Diagnosing COVID-19

It is recognized that certain constellations of symptoms can have predictive value when diagnosing suspected COVID-19, and these can be used in diagnostic and decision-making tools. The current WHO clinical case definition uses “acute respiratory illness,” i.e., fever and a respiratory symptom such as cough or shortness of breath (6). A recent study involving 803 health care workers found that the most frequently reported symptoms among PCR-positive cases were headache, malaise, and myalgia. Anosmia (loss of sense of smell) was 23 times more likely in the PCR-positive group (7). Similarly, another study found that anosmia (excluding those with nasal congestion) had a specificity of around 97% and a sensitivity of 68% for COVID-19 positivity (8). Clinical features can also predict who might become severely ill with COVID-19 or require ICU care. Dyspnea was the best predictor of severe COVID-19 and COPD and cardiovascular diseases were the comorbidities most often found in severely ill patients (9).

## Discussion

A diagnostic algorithm incorporating these symptoms and signs, along with available parameters such as lymphopenia, or bilateral peripheral opacities on plain chest x-ray, could be a way of making care decisions in suspected COVID-19 (a disease which will be around for some time). Advice could then be given on isolation measures and home quarantine for contacts of the case. Also, a patient record capturing this information could be used for clinical case reporting and to assist local disease control efforts. The algorithm should also recognize the diagnostic uncertainty in the outpatient setting in the absence of reliable tests and should therefore signpost actions that also safely manage other likely causes of illness, for example, antibiotics for possible bacterial pneumonia if patients have a fever and/or raised respiratory rate, or antimalarials for patients with a positive malaria parasite test. COVID-19 testing is recommended, but where there is limited access, this does not delay the care and prevention response.

The COVID-19 diagnostic algorithm could be incorporated into guidelines, such as the concise guide we have developed for health facility practitioners that has been made available online (9) as free continuing professional development training modules in Nigeria, promoted through the state health offices. It provides operational guidelines for providing care of patients with possible COVID-19 while continuing other essential care and prevention and focuses on screening, isolation, treatment, and infection prevention and control. The COVID-19 guide was added to a National Institute for Health Research (NIHR)-funded project to test online training of a national guideline for non-communicable diseases. Nurses from 20 primary health care centers in Cross River State received the online training and showed a marked improvement in their pre/post training scores.

In conclusion, rather than the current reality that pretends that timely COVID-19 tests are available while turning a blind eye to ongoing COVID-19 community transmission and failing to protect the people, a concise and user-friendly clinical algorithm should be deployed for COVID-19 suspects in the absence of a diagnostic test. This should use common presenting symptoms and easily accessible investigations to predict likely COVID-19 cases, while acknowledging the diagnostic uncertainty and directing the safe management of diseases with overlapping presentations. This would facilitate the timely provision of appropriate advice on prevention and isolation strategies and provide a case surveillance mechanism to inform local disease control response.

## Author Contributions

The concept and initial draft was provided by JW. Review and edits were done by all authors.

## Conflict of Interest

The authors declare that the research was conducted in the absence of any commercial or financial relationships that could be construed as a potential conflict of interest.
