# Supplementary material for: Clinical Diagnosis and Reporting of COVID-19 in the Absence of Effective Access to Laboratory Testing in Africa
Source: Front Public Health. 2021 May 28;9:645200. doi: 10.3389/fpubh.2021.645200 (PMC8193488; doi:10.3389/fpubh.2021.645200)
Supplement: Supplementary file 1 [file Data_Sheet_1.PDF]

Patient record form for triage of COVID-19 suspects

Name:…………………………………………………………… **Age:**…………. **Sex:**…………**Hospital Number:**…………………….

Home Address:………………………………………………………………… **Telephone number:**………………………….

1. Ask for symptoms

Change of smell or taste, plus one or more of:

- Fever, chills 'hot body' or shivers or temperature > 37.6℃ □Yes □No
- Muscle aches (myalgia), headache, loss of appetite, extreme tiredness □Yes □No
- Sore throat □Yes □No
- Loss of taste or smell □Yes □No
- Persistent cough (dry, new or worse) □Yes □No
- Difficulty breathing/shortness of breath □Yes □No

If ≥ 2 suspect COVID-19 especially so if >2, or if also a contact history in the last 11 days, ask if they:

- Provided direct care to a COVID-19 patient □Yes □No
- Stayed >15 minutes in same closed space with a known COVID-19 case e.g. vehicle, workplace, classroom, household, gatherings □Yes □No

Ask for comorbidity, if any this lowers the threshold to admit, mark and describe:

Chronic disease of lung, kidney, liver, cardio-vascular, sickle cell, or uncontrolled hypertension, diabetes or HIV (not on ARVs), or using steroid tablets or chemotherapy, or obesity (Body Mass Index >40).

If not for admission, say to patients and their family members:

- avoid contact with people coughing and do as in the 'Education' below.
- if more ill, need to seek medical care. If they develop difficulty in breathing – they should go urgently to hospital.

2. Assess the severity of their disease and Act

Use chart below (or CRB65 score) for urgency to admit* (if a pulse oximeter also see NEWS)
Give 1 point for each of:

- Raised respiratory rate (RR, 25 breaths per minute or more).
- Low blood pressure (diastolic ≤60 or less, or systolic <90 mmHg).
- Age 65 years or more.
- Confusion (a new disorientation in person, place or time).

3. Identify 'suspected' If 2 or more symptoms - arrange a test and give infection control advice **Or 'likely' COVID-19** (manage as COVID-19 and arrange a test) if symptoms listed in Step 1 plus:

1. Multiple/specific symptoms e.g. loss of taste or smell, OR also a close contact history OR
2. Patients assessed as severe e.g. RR >30, as above in Step 2 OR

3. Lymphopenia (lymphocytes < 1,100 micro g/L), or lymphocytosis, or a CRP >100 mg/L OR
4. Bilateral opacities on chest X-ray.

Educate on hospital or home isolation for patients with possible COVID-19:

Information on wearing a mask, keeping 2 metre distance, hand washing/ infection control etc.

Home isolation instructions:

1. People with COVID-19 symptoms. To stay home for a minimum of 10 days from when symptoms started (or a positive test) AND until 3 days without fever or respiratory symptoms (other than cough, which may persistent long after being infectious).
2. Family members and other contacts with a COVID-19 case, to stay at home for 14 days from contact, but can end their quarantine if all household members/contacts COVID-19 tests are negative.

Manage COVID-19 Patients according to severity of disease: For all, arrange a test

**Severe**<br><br>Visible difficulty breathing OR<br>Raised respiratory rate (30 breaths/ minute or more) OR<br>Low BP (diastolic ≤60 or less, or systolic <90mmHg) OR<br>Tachycardia (greater than 110) OR<br>New confusion- not clear about person, place or time	**ACTION: urgently** to hospital where oxygen is available.
**Moderate**<br><br>Cough, fever, RR >25 or breathless on exertion - but **not** when speaking and can carry out regular daily tasks	**ACTION:** Give antibiotic if suspect pneumonia, if difficulty breathing – admit to likely COVID ward observation/ assessment.
**Mild flu-like illness:**<br>No moderate or severe COVID-19 symptoms but not difficult breathing or other signs	**ACTION:**<br>Treat and educate on COVID-19 and signs of deterioration

Alternative tools for severity assessment

Severity	Standard	Actions
Severe	CRB65 score = 3-4, or NEWS 3+<br>RR>30/min or pO2 Sats <93%	Urgently transfer to a ward with oxygen<br>Ask for FBC and CXR and throat swab
Moderate	CRB65 total score = 1-2<br>RR>25/min	Send to isolation/observation room/ward for further assessment
Mild	CRB65 total score = 0	Advise and treat, home isolation

NEWS UK national early warning score. If you have an oximeter, use this tool to calculate the score. Rates given for adults (non-pregnant).

NEWS sign	Score			
	0	1	2	3
Respiratory rate	12-20	9 – 11	21-24	≥25 or<br>≤8
Pulse (per minute)	51-90	91-110 or<br>41-50	> 110	≥131 or<br>≤40
Temperature (°C)	36.1-38.0	38.1-39.0 or<br>35.1-36.0	≥39.1	≤35.0
Systolic blood pressure (mmHg)	111 - 219	101-110	91-100	≤90 or<br>≥220
SpO_2 (not on oxygen) by oximeter	≥96	94-95	92-93<br>or on oxygen	≤91
Level of consciousness	Alert			Unresponsive to voice or pain

Or use a similar sepsis/severe illness identification tool. In <5 child e.g. Integrated Maternal, Newborn and Child Health Strategy- 'danger signs'.

Decide the severity of the disease

Severity	Standard
Severe	NEWS with any single 3 or total of 5 and above
Moderate	NEWS total score is 2-4
Mild	NEWS total score is <2
